# Coral Reefs: Beyond Mortality?

**DOI:** 10.1100/tsw.2000.6

**Published:** 2001-04-04

**Authors:** Charles Sheppard

**Affiliations:** Department of Biological Seciences, University of Warwick, Conventry, CV4 7AL, UK

## Abstract

The scale of the collapse of coral reef communities in 1998 following a warming episode (Wilkinson, 2000) was unprecedented, and took many people by surprise. The Indian Ocean was the worst affected with a coral mortality over 75% in many areas such as the Chagos Archipelago (Sheppard, 1999), Seychelles (Spencer et al., 2000) and Maldives (McClanahan, 2000). Several other locations were affected at least as much, with mortality reaching 100% (to the nearest whole number); this is being compiled by various authors (e.g., CORDIO, in press). For example, in the Arabian Gulf, coral mortality is almost total across many large areas of shallow water (Sheppard, unpublished; D. George and D. John, personal communication). The mortality is patchy of course, depending on currents, location inside or outside lagoons, etc., but it is now possible to swim for over 200 m and see not one remaining living coral or soft coral on some previously rich reefs.

This extraordinary mortality has created much scientific interest, but has been largely ignored in the general media. It can only be assumed that the lack of interest is because the problem is perceived to be underwater and far away (Sheppard, in press).

## IS THIS A NATURAL, CYCLICAL, OR MAN-INDUCED EVENT?

Arguments put forward by several government ministers and officials to this author in the last 2 yr are that this is

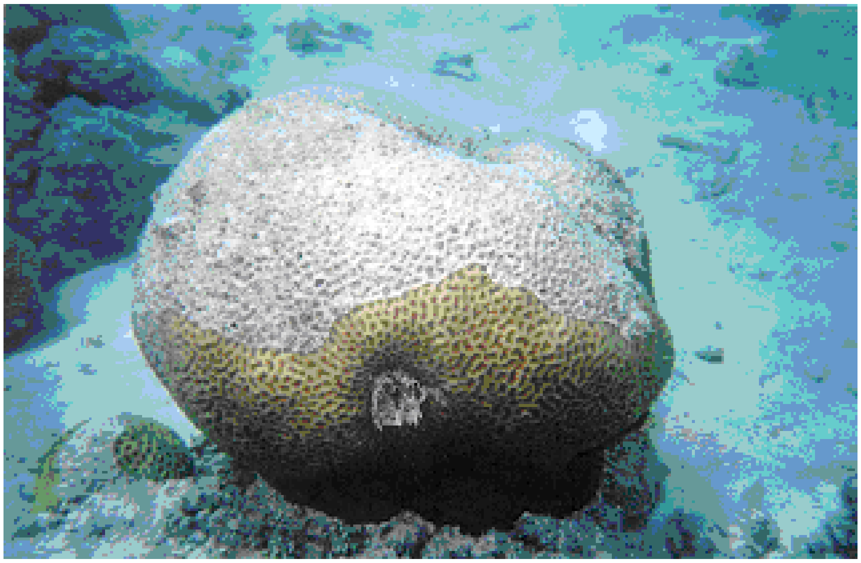

**FIGURE 1**. Example (from Chagos Archipelago) of a partly killed coral in 1999 (a *Goniastrea*). Unusually, part of the coral remains alive. In all such cases, the living tissue lies only in the shaded parts of the colonies; mortality comes not only from warming but also from intense irradiance, including that of UV radiation (see Sheppard 1999).

Previously posted in NewsLab, TheScientificWorld.
© 2000 with author.

probably a natural, cyclical event, so it is not an issue needing much action. However, many of the corals killed were 400-500 yr old (the record known was ~700 yr) so any cycle must have a longer periodicity than this. Indeed, Aronson et al. (2000) show with cores that it has not occurred for at least 3000 yr. Recovery is the important question. Recovery to a fully mature state thus cannot occur before 500 yr or so, though a functioning coral ecosystem may occur sooner. Recruitment of coral spat is indeed underway (Edwards et al., in press), but growth to colony sizes which may more realistically be considered part of a newly emerging 'reef system' currently only occurs at a frequency of about 2 $ha^{-1}$ in the Seychelles (Turner, personal communication). The environment on a dead reef (mobile, dead coral rubble) is not conducive to spat survival, so progress beyond spatfall may be delayed.

Fisheries effects may be mixed. In some places fish populations have collapsed, while in others herbivores are responding well to algae blooms on the dead corals. Possibly both events occur sequentially. It is known that tens of millions of people depend on reef fish for their protein.

It is also evident, based on climate trends, that the high temperatures triggering this event will be near mean values in about 40 yr time! Some recovery claims have supposed that repeat episodes will not occur which set back recovery, but this may not be the case.

## ACTIONS TAKEN

We should expect much research, conservation and planning in response to this massively important event, which seems likely to affect the livelihood of millions of people in the developing world as well as the economy of countries where tourism is important (e.g., Maldives where reef-based tourism provides 40% of national income). However, it is easy to be somewhat cynical about the actions being taken. Over the last decade, when various impacts have resulted in severe damage to perhaps a third of the world's reefs (Bryant et al., 1998), several 'secretariats' and organising bodies have emerged. These are funded in that several agencies contribute to secretariat expenses, but the plans of many seem highly similar and duplicated. Most have produced various action plans and exhortations, but as far as *action* is concerned, the resulting secretariats are then expected to compete for funds to do the research to carry out their plans and answer the same important questions. These questions include crucial ones like: what will be the effects on coastal erosion? and, what will be the effects on fish protein availability? A certain amount of territoriality and competition between the secretariats seems inevitable — at a time when these answers will be increasingly and desperately needed by governments and extensive local communities in the affected areas. Already erosion is taking place (from the 1998 event and earlier coral mortality) and the reef fish availability problem is both severe and persistent (e.g., Linden and Sporong, 1999).

While we know a great deal about the ecology of coral reefs, the present ecological change may be severe enough to create a phase shift (see Hughes, 1994) and remain degraded. At higher taxonomic levels, a healthy coral reef supports the world's most diverse ecosystem, not to mention a high productivity of useable protein, a marked capacity to protect shorelines and ability to generate revenues. Thus it seems prudent to investigate the consequences by signifi-cant direct funding of biological and sociological research in new ways rather than by traditional ones in which there seems to be considerable redundancy. This is complicated, of course, by numerous requirements of donor agencies, 'partnerships', 'co-operation terms' and various political issues. But it may be that this matter cannot wait for niceties - one thing we do know is that bioerosion of the coral structure is well underway! Even before this occurred, many coastal villages of the region were under stress from reduced food security arising from habitat destruction, to the extent that some have even been evacuated in the last 10 yr. The answers to the additional threat imposed by massive coral mortality are needed now.
